# Comfortable Meals in an Institution—Dinner

**Published:** 1912-07-13

**Authors:** 


### INSTITUTIONAL HOUSEKEEPING.

### Comfortable Meals in an Institution—Dinner.

When a well-conducted family or establishment meets for the principal meal of the day it should be taken for granted that each one around the table is healthily hungry. This cannot be the case if the pernicious habit is encouraged of eating between meals. Those who devour buns or cook for themselves secretly cups of Bovril or Benger at eleven o'clock are making a great mistake, and throwing on their digestive systems a burden too heavy to be borne without complaint. A person in good health and under self-control requires only three substantial meals in a day, and when employed in responsible work should have no more thought about her food than the consuming of it demands. If she is worried beforehand about its sufficiency or its punctual service, and is uncomfortable afterwards because of its unsuitability, she is rendered unfit for the real business of her life. Those whose business it is to see that the workers are well nourished have offended against the community and impaired the health and happiness of the nurses or officers unless they can ensure that this condition is fulfilled. When all assemble with cheerful alacrity, certain of a wholesome meal, the dinner-hour is a recreation and refreshment to mind as well as body. Let, then, the chief caterer provide that nothing be wanting within reasonable bounds to the comfort of the simple banquet. Let the food be served hot under covers that are not removed till all are ready. Let enough attendants be ready to serve the company promptly as the appointed hands place the meat on well-heated plates. There should be always a choice offered or two plates handed on the left side to each. Some people cannot digest beef, and still more dislike pork. To argue about such things is ridiculous. Sufficient gravy cannot well be carried in the dish, and it is hotter if served separately and handed with the vegetables. Any suspicion of short commons is most comfortless. If the last to be served receive a plateful of bones and pieces, and find no potatoes are left for them, it is truly a trial of patience. It is best to divide the company in sixes or tens, and not to put all the vegetables in enormous piles, trusting there will be enough to go round. Without this precaution the youngest and hungriest may come short day after day and no notice be taken of it; but if all are well considered there will be relays of vegetable and gravy dishes to suit the dimensions of the party. As to the cooking, enough has been said and is always being said to make it of the first and chief importance to any housekeeper. Two courses only can be served as a rule, and these should suffice; but, because puddings are going out of fashion, it will not do to substitute fruit or fancy cake and pastry in their place at the ordinary mid-day dinner of a number of hard-working persons. This is no place for knick-knacks and tit-bits, which are all that is required where there are four or five courses, and fish or soup have preceded the meat. Nothing can supersede the pudding in a tasty and satisfying form where people are dining with energetic hours of labour before and after the meal. Nevertheless, let not the word "pudding" suggest a heavy and gross compound hard to digest. To many persons suet in any form is a terror, while to many others it is the best means of satisfying the appetite. Let there be then a variety always provided to suit all tastes. The more substantial roly-poly or fruit dumpling should be presented in a palatable shape, with enough jam or fruit to make it acceptable. With this should be a choice of milk pudding and stewed fruit, for numbers of people can only keep well by taking fruit daily. It will be found among a company that half are divided in their choice almost invariably, and there need be no fear of waste by the rejection of one dish provided. When oranges or strawberries are plentiful it makes a pleasing change to introduce these instead of a made-up pudding, but there should be always cheese and biscuits served in this case or the company will not be properly fed. A good housekeeper will consider the weather in planning her menu, and give heating food when it is bitterly cold and cooling dainties when very hot, and her kindness will not be unremarked. Some find themselves better for drinking hot water with their meals, and this need not be impossible to provide; only, let the water be *hot*, not lukewarm, and for this purpose covered fireproof jugs are easy to obtain in large sizes, and do not readily get broken. It is a welcome addition to many if brown bread can be had at dinner-time. Order and comfort and good temper depend largely upon attention to very small details, which, if neglected, cause often loss of appetite and even ill-health.

### Housekeeping Queries.

M. S. *writes:* "*Before I return from my holiday I want to write and tell you how interested I am in the Questions and Answers Column you have lately started. It is most helpful, and many others agree with me. When you have space, could we have a few recipes suitable for convalescent meals—not expensive or elaborate? Like many older nurses, I never received any instruction in cooking during my hospital training, and often find myself handicapped—for books seem so vague. As assistant matron in a convalescent home I find the supervision of cooks a trial.*"

It is very pleasant to receive a letter like this, and we will do our best speedily to furnish the desired recipes for convalescent diet. Since this class of cookery needs to be light, easily digestible, and particularly nourishing in proportion to bulk, it results in precisely the kind of food which best suits busy people. Thus the interests of the convalescents and the staff can be studied side by side.

### NOTICE TO MATRONS.

We are always pleased to answer queries and give recipes which may be asked for by our readers. Let us know if any difficulties are hampering you, and we will do our best to find a remedy. Any successful experiments you may be making in culinary matters would interest a wide circle. We should be specially glad to hear particulars of any scheme for nurses' diet which works out at 6s. a week.

